# The Synergetic Effect of Periodontal Therapy and TNF‐α Inhibitor for the Treatment of Comorbid Periodontitis and Psoriasis

**DOI:** 10.1111/jcpe.14102

**Published:** 2025-04-25

**Authors:** Crystal Marruganti, Carlo Gaeta, Chiara Falciani, Elisa Cinotti, Pietro Rubegni, Mario Alovisi, Nicola Scotti, Andrea Baldi, Cristiana Bellan, Chiara Defraia, Elena Bertaggia, Fabio Fiorino, Silvia Valensin, Erika Bellini, Antonella De Rosa, Filippo Graziani, Francesco D'Aiuto, Simone Grandini

**Affiliations:** ^1^ Unit of Periodontology, Endodontology and Restorative Dentistry, Department of Medical Biotechnologies University of Siena Siena Italy; ^2^ Periodontology Unit, UCL Eastman Dental Institute and Hospital University College London London UK; ^3^ Department of Medical Biotechnologies University of Siena Siena Italy; ^4^ Unit of Dermatology, Department of Medical, Surgical and Neurological Science University of Siena Siena Italy; ^5^ Department of Surgical Sciences, C.I.R. Dental School University of Turin Turin Italy; ^6^ Unit of Anatomical Pathology, Department of Human Pathology and Oncology University of Siena Siena Italy; ^7^ Laboratory of Molecular Microbiology and Biotechnology (LAMMB), Department of Medical Biotechnologies University of Siena Siena Italy; ^8^ Fondazione Toscana Life Sciences Siena Italy; ^9^ Department of Surgery, Unit of Dentistry and Oral Surgery University of Pisa Pisa Italy

**Keywords:** Biologics, periodontal diseases, periodontal therapy, psoriasis, skin diseases

## Abstract

**Aim:**

To assess the adjunctive effect of periodontal therapy on psoriasis‐related outcomes in a combined experimental model of ligature‐induced periodontitis and Imiquimod (IMQ)‐induced psoriasis. Also, this experiment aimed to study the impact of TNF‐α inhibitors on the periodontium.

**Methods:**

Fifty‐six C57/BL6J mice were randomly allocated to seven experimental groups: (a) control group (P–Pso–) with no treatment; (b) periodontitis (P+Pso–) with periodontal therapy; (c) periodontitis (P+Pso–) with TNF‐α inhibitor; (d) psoriasis (P–Pso+) with TNF‐α inhibitor; (e) periodontitis and psoriasis (P+Pso+) with periodontal therapy; (f) P+Pso+ with TNF‐α inhibitor; and (g) P+Pso+ with both periodontal therapy and TNF‐α inhibitor. Samples (maxilla, dorsal skin and blood) were harvested immediately after death. Measures of periodontitis distance between the cemento‐enamel junction and alveolar bone crest (CEJ–ABC) and number of osteoclasts and psoriasis (epidermal thickness and infiltrate cells (per 0.03mm^2^)) severity, as well as systemic inflammation (IL‐6, IL‐17A and TNF‐α) were collected.

**Results:**

In the P+Pso+ group, a significant adjunctive effect of periodontal therapy to TNF‐α inhibitors was found in the reduction of epidermal thickening and inflammatory infiltrate of the dorsal skin (*p* < 0.05). Similarly, treatment with TNF‐α inhibitor resulted in a significant adjunctive effect to periodontal therapy in the reduction of alveolar bone loss (*p* < 0.05). These changes were accompanied by a significant decrease in the circulating levels of IL‐6 and IL‐17A when both periodontal therapy and TNF‐α inhibitor were administered.

**Conclusions:**

The combination of periodontal therapy and TNF‐α inhibitor showed a positive synergetic effect in the treatment of comorbid experimental ligature‐induced periodontitis and IMQ‐induced psoriasis via the reduction of systemic inflammation.

## 1 Introduction

Periodontitis is a highly prevalent, chronic inflammatory disease affecting the tooth‐supporting apparatus (Caton et al. 2018). Its aetiology is multifactorial, and it is usually triggered by an aberrant immune response to a dysbiotic dental biofilm in susceptible individuals due to systemic or environmental factors (Curtis, Diaz, and Van Dyke 2020; Loos and Needleman 2020; Marruganti, Baima, Grandini, et al. 2023; Marruganti et al. 2022; Marruganti, Gaeta, Romandini, et al. 2024; Marruganti, Romandini, et al. 2023). At a local level, if periodontitis is left untreated, it eventually leads to tooth loss as well as masticatory and aesthetic dysfunctions (Tonetti, Greenwell, and Kornman 2018). The inflammatory burden of periodontitis was also found to exert negative effects on systemic health, mainly via the bacterial translocation from the oral cavity to the blood stream or through the induction of a state of low‐grade systemic inflammation (LGSI) (Hajishengallis and Chavakis 2021). As such, severe periodontitis has been associated with an increased risk of several systemic diseases (Eezammuddeen, Vaithilingam, and Hassan 2023; Fan et al. 2023; Marruganti, Baima, Aimetti, et al. 2023; Marruganti, Suvan, et al. 2023; Zamora‐Pasadas et al. 2022; Zhao et al. 2022), and its treatment has been demonstrated to have positive effects also on general health, through the reduction of glycated haemoglobin and blood pressure levels, among others (Czesnikiewicz‐Guzik et al. 2019; D'Aiuto et al. 2018; Kamata and Tada 2020).

Psoriasis is a chronic inflammatory skin disorder characterized by the rapid proliferation of keratinocytes and leading to the formation of thick scaly patches on the skin, severely impacting the quality of life of those affected by the disease (Kamata and Tada 2020). While psoriasis manifests primarily as a skin disorder, it was also found to have detrimental systemic implications mainly through the activation of the LGSI axis (Valdimarsson et al. 2004). Depending on the disease severity, the treatment of psoriasis may include topical or systemic medications aiming to reduce skin inflammation and slow down keratinocyte proliferation. According to the treatment guidelines, biological medications, such as tumour necrosis factor α (TNF‐α) inhibitors, represent the most common treatment for severe plaque psoriasis (Mrowietz et al. 2011). However, the available treatments are often insufficient (Tokuyama and Mabuchi 2020).

The association between periodontitis and psoriasis has been investigated in clinical and preclinical studies, suggesting a bidirectional association between the two diseases, mediated by shared inflammatory pathways as well as numerous common risk factors (Kurd and Gelfand 2009; Marruganti, Gaeta, Romandini, et al. 2024). Moreover, previous randomized clinical trials have demonstrated a potential beneficial impact of periodontal therapy in the clinical reduction of the extent and severity of psoriasis (Marruganti, Romandini, et al. 2024). However, no mechanistic studies are available about the impact of periodontal treatment on the histological outcomes of psoriasis and whether this retains a significant adjunctive effect to the use of biological medications. The main study hypothesis regards the potential significant additional effect of periodontal therapy to TNF‐α inhibitor in reducing the severity and extent of psoriasis via the reduction of systemic inflammation in a combined experimental model of periodontitis and psoriasis. Furthermore, little is still known about the effect of biological medications on the periodontium. The main aim of this preclinical investigation was to assess the adjunctive impact of periodontal therapy on psoriasis‐related outcomes in a previously tested (Marruganti, Gaeta, Falciani, et al. 2024) combined experimental model of ligature‐induced periodontitis and Imiquimod (IMQ)‐induced psoriasis. Secondarily, this experiment aimed to study the impact of TNF‐α inhibitors on the periodontium.

## 2 Materials and Methods

This preclinical study was designed following the modified ARRIVE guidelines for preclinical research (Vignoletti and Abrahamsson 2012) and the European Union regulations (European Communities Council Directive 86/609/EEC). This study is the second part to the protocol that was performed at the Toscana Life Sciences (Siena, Italy) animal facilities and approved by the local Animal Welfare Body and the Italian Ministry of Health (468/2021‐PR). Results from the first part of the protocol were recently published (Marruganti, Gaeta, Falciani, et al. 2024).

### 2.1 Study Design

**FIGURE 1:**
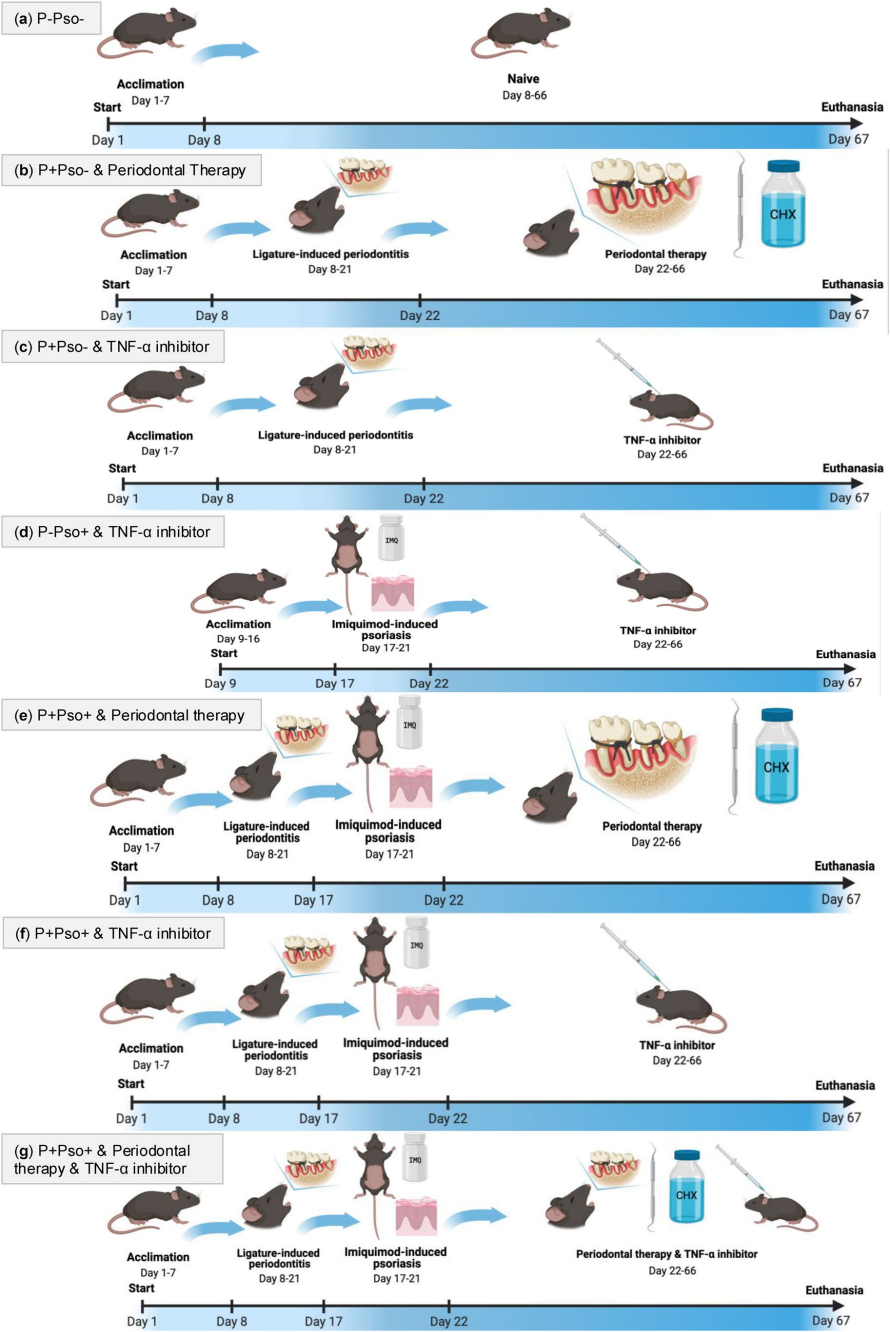
(a–g) Flow chart describing the experimental phases for each group. Figure created with BioRender.com.

Fifty‐six male C57/BL6J mice (Charles River Laboratories, Calco, Italy) (20–30 g) aged 8–12 weeks were kept in constant conditions and fed ad libitum for 7 days prior to the experiments (acclimation period) (Figure 1). Mice were randomly allocated to seven experimental groups (*n* = 8) resulting from the different combinations of induction of periodontitis (P) and psoriasis (Pso), as well as the different combinations of periodontal and psoriasis therapy (carried out with TNF‐α inhibitors): (a) control group (P–Pso–) with no treatment; (b) periodontitis (P+Pso–) with periodontal therapy; (c) periodontitis (P+Pso–) with TNF‐α inhibitor; (d) psoriasis (P–Pso+) with TNF‐α inhibitor; (e) periodontitis and psoriasis (P+Pso+) with periodontal therapy; (f) P+Pso+ with TNF‐α inhibitor; (g) P+Pso+ with both periodontal therapy and TNF‐α inhibitor.

#### 2.1.1 Ligature‐Induced Periodontitis and IMQ‐Induced Psoriasis Models

Similar to the previous study published by this group (Marruganti, Gaeta, Falciani, et al. 2024), a ligature‐induced model of periodontitis was used. Anaesthesia was administered through an intraperitoneal injection of a mixture containing 66.7 mg/kg of ketamine and 6.7 mg/kg of xylazine. Afterwards, sterile silk sutures (5–0) were tied around the right and left second (M2) and third (M3) maxillary molars and retained in place for 14 days (Marchesan et al. 2018).

For the induction of psoriasis, each mouse was administered a topical dose of 62.5 mg of commercially available 5% IMQ cream (Aldara; 3M Pharmaceuticals; Maplewood, MN, USA) on their shaved dorsal skin for five consecutive days (Marruganti, Gaeta, Falciani, et al. 2024; van der Fits et al. 2009).

#### 2.1.2 Periodontal Therapy

After the induction of periodontitis, periodontal therapy was administered using hand instruments (type 1–2 micro‐mini Gracey curettes; HuFriedy, Chicago, USA) under anaesthesia as described above. In addition, chemical plaque control was achieved through oral applications of 50 μL chlorhexidine 0.12% every 2 days until the 45th day after the start of the treatment (Lübcke et al. 2019; Queiroz‐Junior et al. 2012).

#### 2.1.3 TNF‐α Inhibitor

After the induction of either periodontitis or psoriasis, 10 mg/kg of an anti‐TNF‐α monoclonal antibody (Adalimumab; AbbVie, Chicago, USA) was administered via intraperitoneal injection every 2 days until the 45th day after the induction of periodontitis/psoriasis (Queiroz‐Junior et al. 2012).

#### 2.1.4 Tissue Specimens

At the end of the study period (day 67; Figure 1), the mice were euthanized using 3.5% isoflurane inhalation followed by cervical dislocation. Immediately after euthanasia, samples of the maxillae, dorsal skin and blood were collected. Blood was drawn via cardiac puncture, then treated with ethylenediaminetetraacetic acid (EDTA, 1% w, 1 vol. EDTA per 50 vol. blood) to prevent coagulation and finally centrifuged at room temperature for 15 min to isolate plasma. Moreover, the mandible was separated from the skull, and the dorsal skin was harvested. All specimens, except for the dorsal skin, were stored at −80°C (Marruganti, Gaeta, Romandini, et al. 2024).

### 2.2 Study Outcomes

The study outcomes include radiographic alveolar bone loss (both linear and volumetric), histological parameters of the palatal tissues and the dorsal skin, as well as markers of systemic inflammation. Further details on the outcome assessment methods are described elsewhere (Marruganti, Gaeta, Romandini, et al. 2024). All analyses of study outcomes were performed masked to study groups.

#### 2.2.1 Alveolar Bone Loss

Following euthanasia, palatal tissue samples were fixed in 4% paraformaldehyde (PFA) in phosphate‐buffered saline (PBS; pH 7.4) for 24 h. After fixation, samples were decalcified in 25% EDTA for 2 weeks, with the solution being changed three times per week and interrupted by 1 h soaking in distilled water. The samples were then drained in an ascending concentration of alcohol, embedded in paraffin and cut in 4‐μm‐thick slices. Samples were also examined with high‐resolution scans using micro‐computed tomography (micro‐CT) (SkyScan 1172 Micro‐CT, Bruker, Billerica, MA, USA) and image reconstruction (NRecon, Bruker). Segmentation was performed using a commercial software (Mimics 20.0, Materialise, Ann Arbor, MI, USA) to create a 3D rendering of the entire palatal tissues. The cemento‐enamel junction–alveolar bone crest (CEJ–ABC) distance was measured by a single examiner who had been previously calibrated. Details on the examiner's calibration process are reported elsewhere (Marruganti, Gaeta, Falciani, et al. 2024). The CEJ–ABC distance, measured in millimetres, was used to measure the alveolar bone loss. It was obtained by taking four measurements for the mesial peak (MB1, MB2, MP1, MP2) and four for the distal peak (DB1, DB2, DP1, DP2) of each molar from the buccal to the palatal aspect of each tooth in order to map the interproximal bone profile. The mean of the M2 and M3 measurements taken from both sides (right and left) was used as the overall CEJ–ABC distance. The larger the CEJ–ABC distance, the higher the alveolar bone loss.

Furthermore, bone volumetric analysis was performed on each sample by identifying the volume of interest (VOI) in the area between M2 and M3 and manually excluding the palatal bone to reduce the impact of any anatomical variables among the samples (Geomagic Qualify Studio 12, 3D Systems). The final volume calculation was then expressed as bone volume/total volume (%) (Marruganti, Gaeta, Falciani, et al. 2024).

#### 2.2.2 Histological Outcomes

##### 2.2.2.1 Palatal Tissues

Maxillary samples were demineralized and embedded in paraffin, and 4‐μm‐thick sagittal sections were prepared around the region of interest (M2–M3) for histological and immunohistochemical analysis. These analyses were conducted by a previously calibrated single examiner, blinded to group allocation. Osteoclasts were stained using the tartrate‐resistant acid phosphatase (TRAP) kit (387A‐1KTF; Sigma Chemical Co.) according to the manufacturer's instructions; haematoxylin Gill 3 (Sigma‐Aldrich) was used for counterstaining. For each sample, four fields of 1.13 mm^2^ each (at 20× magnification) were examined, and the concentration of osteoclasts was determined by counting the number of TRAP‐positive multinucleated cells in each field. For each animal, the mean concentration of osteoclasts across these four fields was then calculated (Marruganti, Gaeta, Falciani, et al. 2024).

##### 2.2.2.2 Dorsal Skin

Mice were perfused with 4% PFA, and the dorsal skin was then fixed in 10% neutral‐buffered formalin, processed and embedded in paraffin blocks. Sections of 4 μm thickness were obtained using a rotary microtome, stained with haematoxylin and eosin (H&E; Muto Pure Chemicals Co. Ltd.; Tokyo, Japan) and then examined with a digital microscope (OMAX 40X 2500X LED, ZEISS, Germany). Epidermal thickness was calculated as the epidermal area (μm^2^) divided by the basal membrane length (μm), measured in two separate fields, each covering 1.13 mm^2^ per sample; the mean value was then considered (Ju et al. 2019). The extent of inflammatory infiltrate in the dermis of the entire dorsal skin of the mice was quantified as the number of infiltrated cells per 0.03 mm^2^. All acquisitions, measurements and histological analyses were performed using the digital microscope software (ZEN Blue 3.4 software, ZEISS). Each measurement was performed twice by a single blinded examiner and the mean value recorded (Marruganti, Gaeta, Falciani, et al. 2024).

#### 2.2.3 Systemic Inflammation

The concentrations of IL‐6, IL‐17A and TNF‐α in blood serum samples were measured using Luminex immunoassay (Luminex Assays, Thermo Fisher Scientific, Waltham, MA). Analytes were detected (BioPlex pro Mouse Cytokine Group 1, BioRad, Hercules, CA) and analysed (Bio‐Plex Magpix Multiplex Reader, Bio‐Rad) with the BioPlex technology according to manufacturer's instructions. Cytokine concentrations (pg/mL) were calculated in duplicate based on the standard curve for each plate and analysed with the ad hoc software (BioPlex Manager 6.2 software, BioRad). Both intra‐ and inter‐assay coefficients of variation were below 6% (Marruganti, Gaeta, Falciani, et al. 2024).

### 2.3 Data Analyses

All analyses were performed using a statistical software (Stata/MP, version 18, StataCorp LLC, College Station, TX) setting the significance level at 5%. The sample size calculation was based on the predicted variance in means (0.3) of the primary outcome measure, that is, the number of infiltrate cells/0.03mm^2^ in the dorsal skin, which was derived from the results obtained in a previous publication (Marruganti, Gaeta, Romandini, et al. 2024). Considering a power of 80% and an error alfa of 0.05, the resulting sample size considering seven groups with equal weight was eight animals per group (*n* = 56).

Variables are reported as mean with standard deviations (SDs) or as medians with interquartile ranges (IQRs). Depending on data distribution, inter‐group and pairwise comparisons were performed with ANOVA followed by post hoc Tukey's tests for normally distributed variables, and with Kruskal–Wallis followed by post hoc Dunn's tests in case of non‐normal distribution.

## 3 Results

### 3.1 Study Sample

A total of eight animals were allocated to each experimental group (*N* = 56). Two mice died during the administration of the anaesthesia required to place the ligatures in group P+ Pso– and TNF‐α inhibitor (*n* = 7) and in group P+Pso+ and periodontal therapy (*n* = 7). One mouse died during the administration of the second anaesthesia required to perform mechanical periodontal therapy in group P+Pso+ with periodontal therapy and TNF‐α inhibitor (*n* = 7). The final sample analysed included 53 animals.

### 3.2 Alveolar Bone Levels

**FIGURE 2:**
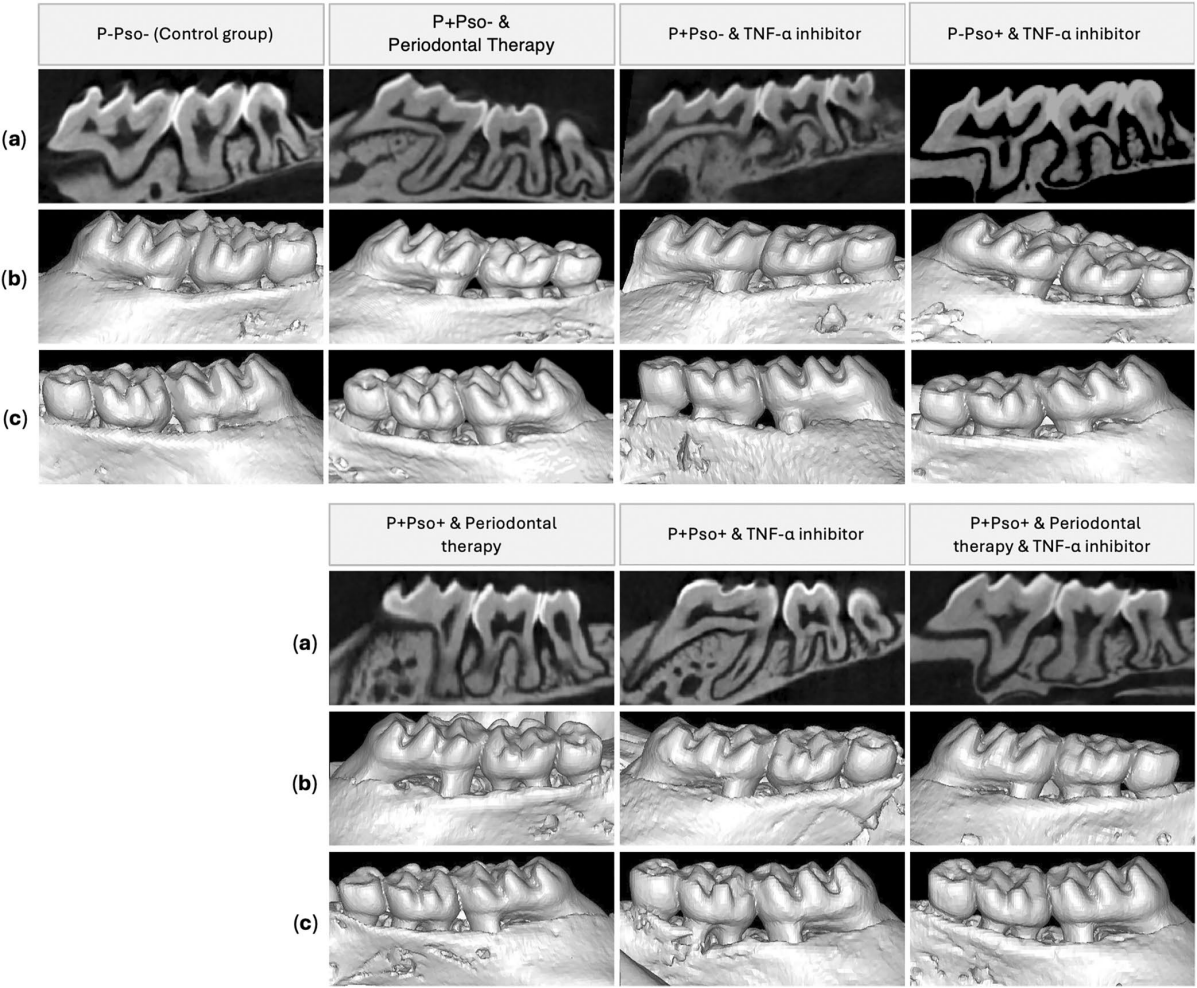
Representative bidimensional (a) and sagittal 3D views of the maxillary molars for each group of the buccal right (b) and left side (c).

**FIGURE 3:**
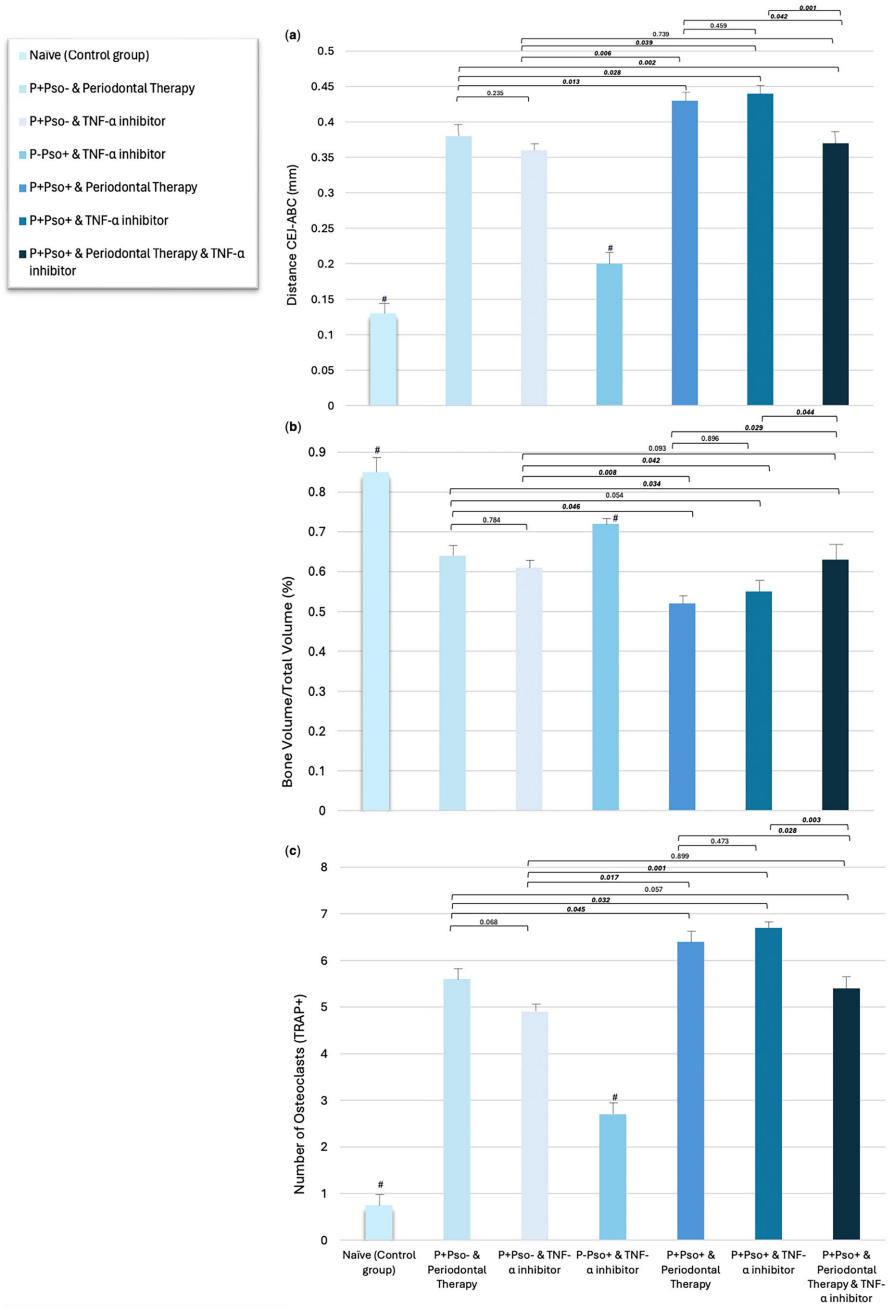
(a) Histogram showing the mean distance between the cemento‐enamel junction and the alveolar bone crest for each group (error bars indicate 95% confidence intervals). (b) Histogram showing the mean values of bone volume/total volume (%) for each group (error bars indicate 95% confidence intervals). (c) Histogram showing the mean number of osteoclasts (TRAP+ cells) in the gingival tissues (error bars indicate 95% confidence intervals). For all histograms, groups indicated with the symbol # are significantly different compared to all other groups (*p* < 0.05).

Representative bidimensional and three‐dimensional views of the maxillary molars for each group are shown in Figure 2. At day 67, the control group (P–Pso–) presented the lowest mean CEJ–ABC distance (0.1 ± 0.04 mm) when compared to the other groups, while the groups with P+Pso+ and periodontal therapy (0.4 ± 0.05 mm) and P+Pso+ and TNF‐α inhibitor (0.4 ± 0.03 mm) showed the highest values, with statistically significant differences when compared to the P+Pso+ with both periodontal therapy and TNF‐α inhibitor (0.3 ± 0.02 mm; *p* < 0.05; Figure 3a). Moreover, the P+Pso+ group with both periodontal therapy and TNF‐α inhibitor showed a significantly lower CEJ–ABC distance when compared to the P+Pso– group with periodontal therapy alone (*p* = 0.002). Along the same lines, periodontal therapy and TNF‐α inhibitor demonstrated comparable bone levels in the P+Pso– groups (*p* = 0.235) (Figure 3a). Bone volume analyses reported consistent results, with the control group (P–Pso–) showing the highest mean value and the P+Pso+ group with periodontal therapy showing the lowest mean value of bone volume (Figure 3b).

### 3.3 Histological Outcomes

#### 3.3.1 Palatal Tissues

At day 67, the control group (P–Pso–) presented the lowest bone resorption (0.7 ± 0.20 mm) when compared to the other groups, while the groups with P+Pso+ and periodontal therapy (6.4 ± 0.22 mm) and P+Pso+ and TNF‐α inhibitor (6.7 ± 0.17 mm) showed the highest values, with statistically significant differences when compared to the P+Pso+ group with both periodontal therapy and TNF‐α inhibitor (5.4 ± 0.28 mm; *p* < 0.05; Figure 3c). Moreover, the P+Pso+ group with both periodontal therapy and TNF‐α inhibitor showed a smaller number of osteoclasts when compared to the P+Pso– group with periodontal therapy alone, although not significant (*p* = 0.057). Along the same lines, periodontal therapy and TNF‐α inhibitor demonstrated a comparable number of osteoclasts as the P+Pso– groups (*p* = 0.058) (Figure 3c).

#### 3.3.2 Dorsal Skin

**FIGURE 4:**
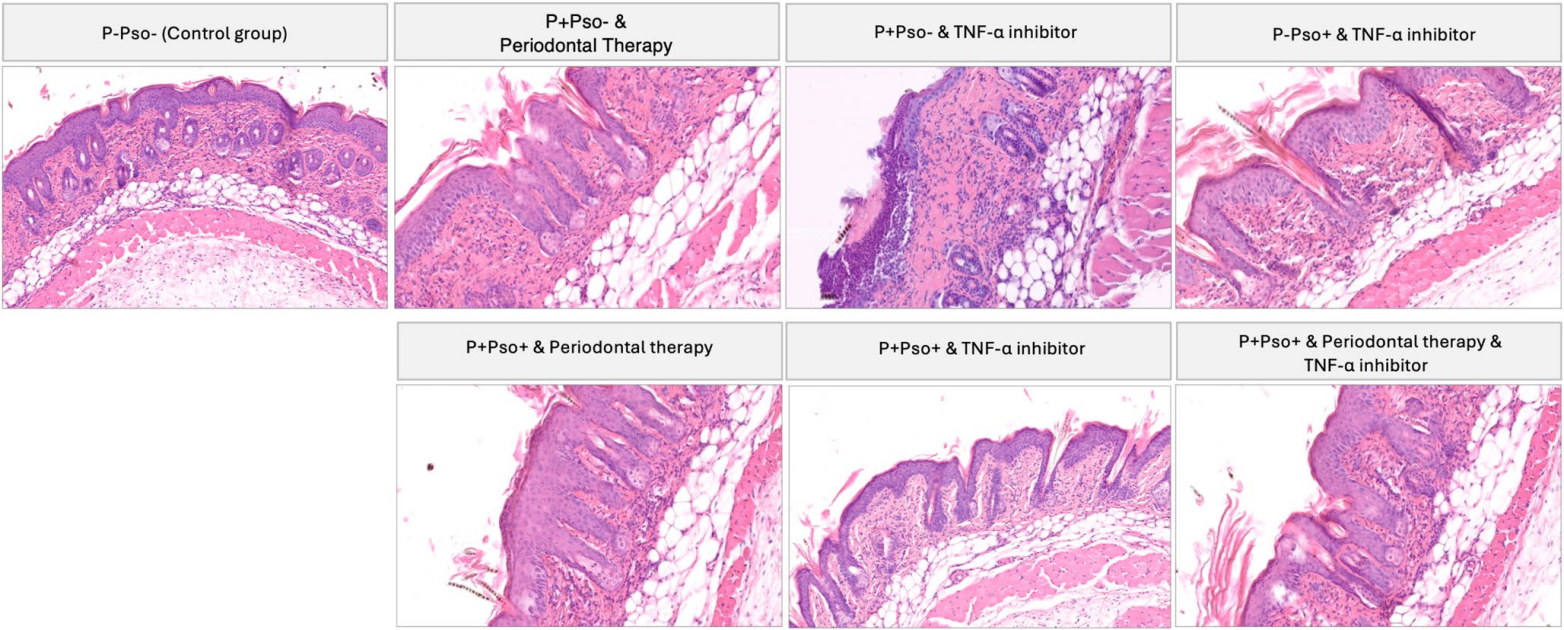
Representative H&E‐stained dorsal skin sections for each group.

**FIGURE 5:**
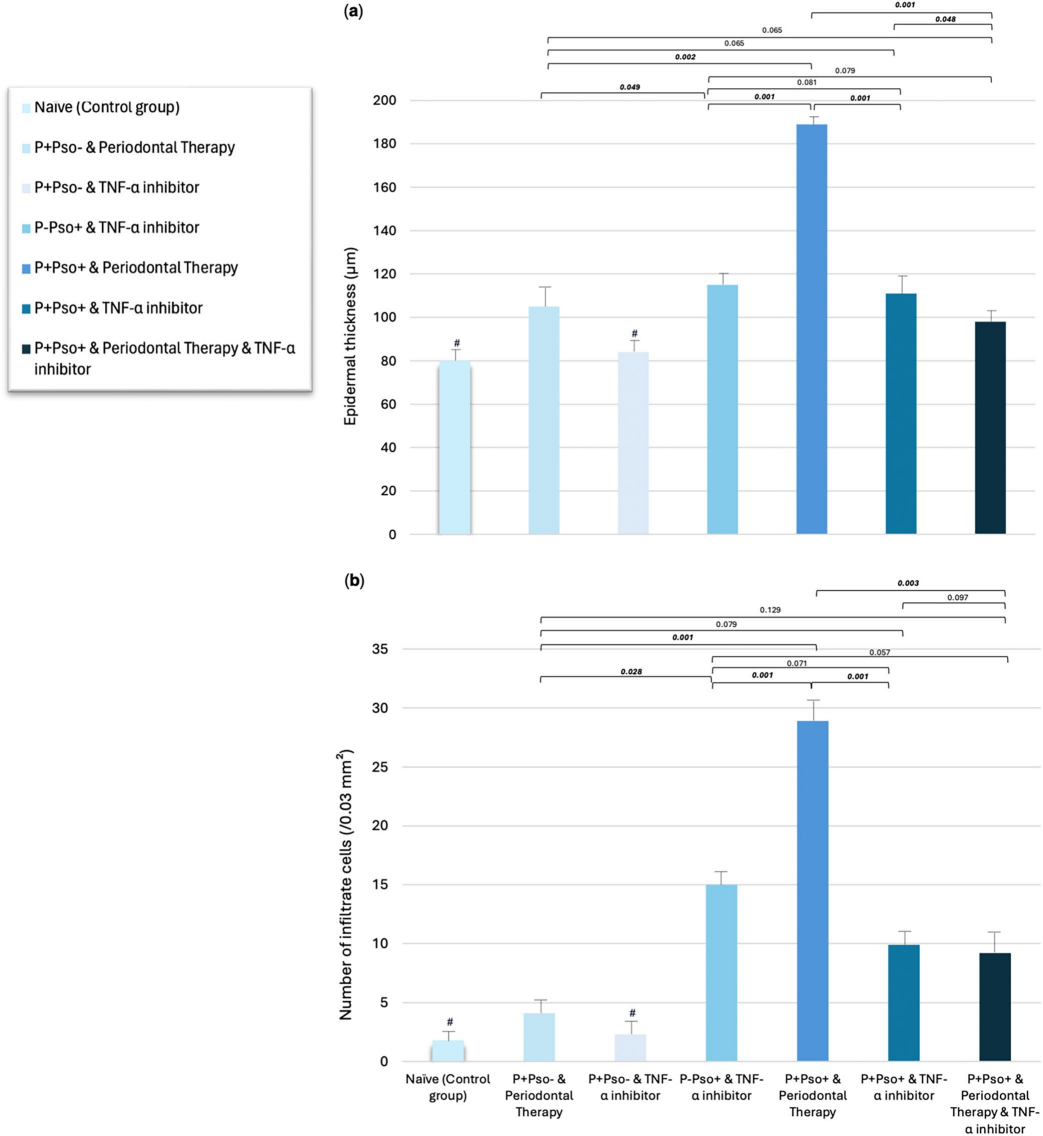
(a) Histogram showing the mean epidermal thickness and the (b) number of infiltrate cells for each group (error bars indicate 95% confidence intervals). For all histograms, groups indicated with the symbol # are significantly different compared to all other groups (*p* < 0.05).

Representative H&E‐stained dorsal skin sections for each group are shown in Figure 4. The lowest value of epidermal thickness was recorded in the control group (80.0 ± 12.3 mm), while the highest was in the P+Pso+ group with periodontal therapy alone (189.1 ± 12.4 mm), with statistically significant differences when compared to the P+Pso+ group with both periodontal therapy and TNF‐α inhibitor (98.3 ± 18.9 mm; *p* < 0.05; Figure 5a). Moreover, the P+Pso+ group with both periodontal therapy and TNF‐α inhibitor showed a lower epidermal thickness than the P+Pso– group with periodontal therapy alone, although not significant (*p* = 0.065). Along the same lines, TNF‐α inhibitor demonstrated significancantly lower epidermal thickening compared to periodontal therapy in the P+Pso– groups (*p* < 0.05) (Figure 5a).

The lowest number of infiltrate cells was recorded in the control group (1.8 ± 0.5 mm), while the highest was in the P+Pso+ group with periodontal therapy alone (28.9 ± 2.1 mm), with statistically significant differences when compared to the P+Pso+ group with both periodontal therapy and TNF‐α inhibitor (9.2 ± 18.9 mm; *p* = 0.003; Figure 5b). Moreover, the P+Pso+ group with both periodontal therapy and TNF‐α inhibitor showed a comparable number of infiltrate cells as the P+Pso– group with periodontal therapy alone (*p* = 0.129). Along the same lines, TNF‐α inhibitor resulted in a significancantly smaller inflammatory infiltrate when compared to periodontal therapy in the P+Pso– groups (*p* < 0.05) (Figure 5a).

### 3.4 Systemic Inflammation

**FIGURE 6:**
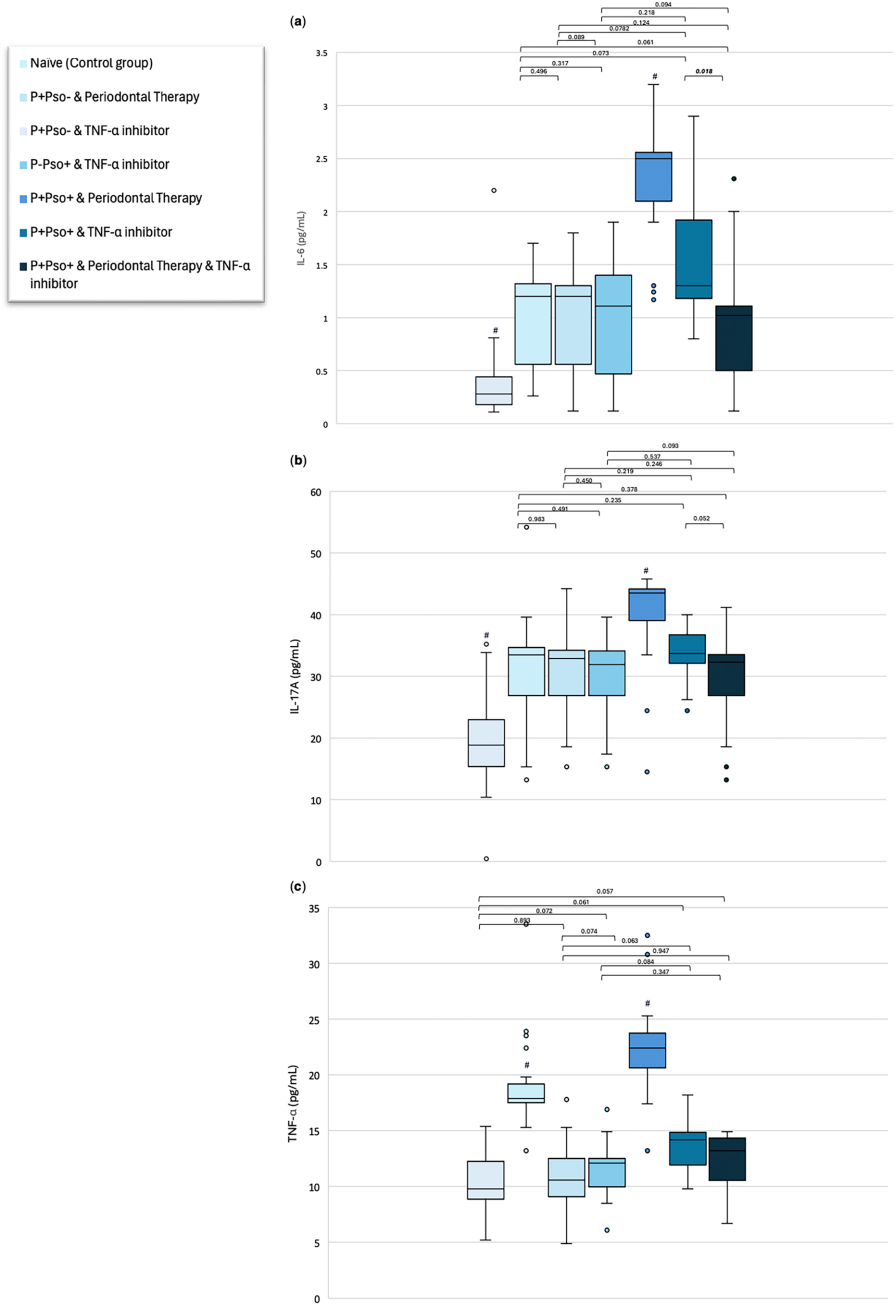
Boxplots showing IL‐6 (a), IL‐17A (b) and TNF‐a (c) levels for each group (boxes and bars indicate the median value and interquartile range, respectively). For all boxplots, groups indicated with the symbol # are significantly different compared to all other groups (*p* < 0.05).

All experimental groups exhibited statistically significantly higher concentrations of IL‐6, IL‐17A and TNF‐α than the control group (*p* < 0.05; Figure 6a–c). IL‐6, IL‐17A and TNF‐α reached their highest levels in the P+Pso+ group with periodontal therapy, while their lowest concentrations were recorded for the control group. The P+Pso+ group with both periodontal therapy and TNF‐α inhibitor showed lower levels of systemic inflammation than the P+Pso+ group with periodontal therapy alone and TNF‐α inhibitor alone; these comparisons reached statistical significance in the former group for all the considered markers but in the latter group for IL‐6 only. Moreover, the P+Pso+ group with both periodontal therapy and TNF‐α inhibitor demonstrated comparable levels of IL‐6 and IL17A as the P+Pso– and P–Pso+ groups irrespective of their treatment allocation (*p* > 0.05; Figure 6a–c).

## 4 Discussion

In this experiment, periodontal therapy and TNF‐α inhibitor showed a synergetic effect in the treatment of comorbid experimental ligature‐induced periodontitis and IMQ‐induced psoriasis. Both periodontal therapy and TNF‐α inhibitor were found to be effective in reducing alveolar bone resorption with a similar magnitude of effect. The treatment with TNF‐α inhibitor had a significant adjunctive effect to periodontal therapy in the reduction of alveolar bone loss in the combined model of periodontitis and psorasis. In the same combined model, a similar adjunctive effect was shown by periodontal therapy in the reduction of epidermal thickening and inflammatory infiltrate of the dorsal skin. Moreover, in this combined model of periodontitis and psoriasis, circulating levels of inflammatory markers further decreased when both periodontal therapy and TNF‐α inhibitor were administered, thus suggesting a potential association linking the two diseases in humans.

Experimental periodontitis and psoriasis, as well as their combination, were successfully induced in all study groups as per group allocation, as demonstrated by the higher alveolar bone resorption, larger inflammatory infiltrate of the dorsal skin and higher concentration of serum inflammatory markers compared to the control group. Moreover, animals with experimental psoriasis treated with TNF‐α inhibitor showed higher alveolar bone loss when compared to the control group (naive); as well, animals with experimental periodontitis treated with periodontal therapy showed a higher tendency towards epidermal thickening and a larger inflammatory infiltrate of the dorsal skin.

In this experiment, the treatment with TNF‐α inhibitor was found to be as effective in reducing alveolar bone loss as periodontal therapy. This result is consistent with previous preclinical and clinical studies showing the benefits of TNF‐α inhibitors on the periodontium (Cao et al. 2017; De Vries et al. 2019; Fabri et al. 2015; Mirastschijski et al. 2019; Zamri and de Vries 2020). From a clinical standpoint, recent meta‐analytical data showed that TNF‐α inhibitors led to a significant reduction in bleeding on probing (BoP) after 6 weeks of therapy, as well as significant reductions in probing pocket depth (PPD) and clinical attachment level (CAL) starting from 3 months after treatment (Fabri et al. 2015; Zamri and de Vries 2020). These clinical improvements may be due to a reduction in all TNF‐α induced cellular processes. In particular, TNF‐α inhibitors may cause the initial decrease in clinical BoP due to the reduction in vasodilation and vessel permeability for leukocytes. In turn, these processes may lead to a decrease in the leukocyte‐induced proteolytic activity and osteoclast signalling (Cao et al. 2017; De Vries et al. 2019; Mirastschijski et al. 2019), thus potentially explaining the improvements of clinical periodontal parameters.

This experiment represents the first mechanistic study investigating the significant positive adjunctive effect of periodontal therapy for the treatment of psoriasis in a combined model of experimental periodontitis and psoriasis. In particular, periodontal therapy was shown to induce further decrease in epidermal thickness and inflammatory infiltrate of the dorsal skin when performed as adjunctive to TNF‐α inhibitors. These results are consistent with two previous randomized clinical trials demonstrating a significant reduction in the extent and severity of plaque psoriasis, as measured with the psoriasis area and severity index (PASI) and body surface area (BSA), after non‐surgical periodontal therapy when compared to no treatment (i.e. untreated periodontitis) (Marruganti, Gaeta, Romandini, et al. 2024; Ucan Yarkac, Ogrum, and Gokturk 2020). Specific biological mechanisms may potentially explain the results obtained. Firstly, the changes in the oral microbiome after periodontal therapy may lead to a reduction in the bacterial translocation from the oral cavity to the skin. Indeed, recent evidence has highlighted how the psoriasis onset can be triggered by specific changes to the skin microbiome, which, in turn, was found to be strongly influenced by alterations of the gut microbiota (Fry et al. 2013; Rademaker et al. 2019). Hence, given the significant changes in the gut microbiota composition following periodontal therapy (Baima et al. 2024), the existence of a reciprocal influence of the oral and skin microbiota can be hypothesized and may be at the root of the significant amelioration of psoriasis following periodontal therapy.

An alternative pathway is represented by the significant reduction in the inflammatory burden following periodontal therapy as well as the immunological shift from a dominance of innate immunity towards a dominance of acquired immune, which was found to potentially favour a state of quiescient psoriasis (Christophers and van de Kerkhof 2019; Hajishengallis and Chavakis 2021). Indeed, an additional decrease in systemic inflammatory markers following the combination of periodontal therapy and TNF‐α inhibitor was witnessed in this experiment, thus supporting the existance of this pathway.

Some limitations should be considered when interpreting these findings, including the lack of assessment of the skin, gut and oral microbiota. Although ligature‐induced periodontitis might cause more significant trauma to periodontal tissues compared to other experimental models, such as oral gavage with *P. gingivalis* (Klausen 1991), and despite the possibility that the ligatures around the molars were not stable throughout the experimental period and especially after periodontal therapy (Marchesan et al. 2018), this method achieves substantial biofilm accumulation, bone loss and robust host inflammatory responses in a shorter timeframe (de Molon et al. 2016; Hajishengallis and Chavakis 2021). The direct IMQ‐induced model of psoriasis was chosen over other models, such as genetically engineered animals and cytokine injections, because of its recognized translational potential, convenience and repeatability in inducing psoriasiform skin inflammation (Swindell et al. 2017). However, this model may mimick only limited aspects of human psoriasis and may potentially cause unintended systemic effects due to IMQ ingestion during the experiment, which could have influenced the results obtained (Gangwar, Gudjonsson, and Ward 2022). Moreover, other systemic inflammatory markers as well as additional histological measures of inflammation could have been relevant to further corroborate the findings obtained. It is also important to note that the P+Pso– and P+Pso+ as well as the groups where periodontal therapy was performed may have been influenced by the general anaesthesia used, while the P–Pso+ and P+Pso+ groups may have experienced discomfort due to the psoriatic lesions. Nevertheless, any stressors or discomfort was mitigated using environmental enrichment strategies for the animals (Vachon et al. 2013). Furthermore, multiple comparisons among study groups might have reduced the power of some of the estimates obtained. Despite these considerations, a rigorous experimental methodology and analysis was employed (Marchesan et al. 2018; Marruganti, Gaeta, Romandini, et al. 2024; Swindell et al. 2017).

## 5 Conclusion

Evidence from this preclinical model suggests that periodontal therapy and TNF‐α inhibitor exert a positive synergetic effect in the treatment of comorbid experimental ligature‐induced periodontitis and IMQ‐induced psoriasis. In particular, TNF‐α inhibitor may successfully reduce alveolar bone loss, and, vice versa, periodontal therapy may contribute to the reduction of the signs of psoriasiform skin inflammation. These changes were accompanied by reductions in systemic inflammatory markers, thus further supporting the common inflammatory pathways between the two diseases. Additional experimental and clinical studies should be performed to further investigate the effects of the combined treatment of both diseases. The significant adjunctive effect of periodontal therapy to TNF‐α inhibitor for the treatment of psoriasis supports the importance of the early diagnosis and treatment of periodontitis in individuals affected by comorbid psoriasis. In turn, TNF‐α inhibitor was found to have a beneficial effect not only for the successful management of psoriasis but also for the amelioration of periodontal outcomes. These findings should further encourage the teamwork between dermatologists and periodontists for the joint management and treatment of life‐long chronic conditions such as periodontitis and psoriasis, and hence a bidirectional referral pathway between dermatologists and periodontists is being proposed here. Additional experimental and clinical studies should be performed to further investigate the effects of the combined treatment of both diseases.

## Author Contributions

Crystal Marruganti contributed to study conception, study design, data analysis, data collection and manuscript drafting. Carlo Gaeta contributed to data analysis, data interpretation and manuscript drafting. Chiara Falciani, Erika Bellini, Silvia Valensin, Elena Bertaggia and Antonella De Rosa contributed to setting up the experimental model. Elisa Cinotti and Pietro Rubegni contributed to study conception, study design and manuscript drafting. Mario Alovisi and Nicola Scotti contributed to micro‐CT analyses. Andrea Baldi contributed to bone volumetric analyses in micro‐CT. Cristiana Bellan and Chiara Defraia contributed to histological analyses. Fabio Fiorino contributed to cytokine analyses. Francesco D'Aiuto and Filippo Graziani contributed to data analysis, data presentation and manuscript drafting. Simone Grandini contributed to study conception, study design and manuscript drafting.

## Ethics Statement

The study was ethically approved by the local Animal Welfare Body and the Italian Ministry of Health (468/2021‐PR).

## Conflicts of Interest

The authors declare no conflicts of interest.

## Data Availability

The data that support the findings of this study are available upon reasonable request from the corresponding author. The data are not publicly available due to privacy or ethical restrictions.
